# Comparison of Chest High-Resolution Computed Tomography Findings in Patients with Anti-Melanoma Differentiation-Associated Gene 5 Antibody-Positive and Antibody-Negative Progressive Pulmonary Fibrosis with Polymyositis/Dermatomyositis

**DOI:** 10.3390/jcm14051601

**Published:** 2025-02-27

**Authors:** Noboro Sato, Takuya Kotani, Mitsuhiro Koyama, Shogo Matsuda, Aya Sakamoto, Yoshihiro Shou, Katsumasa Oe, Tohru Takeuchi, Keigo Osuga

**Affiliations:** 1 Department of Diagnostic Radiology, Osaka Medical and Pharmaceutical University, Takatsuki 569-8686, Osaka, Japanmitsuhiro.koyama@ompu.ac.jp (M.K.); 2 Department of Radiology, Tominaga Hospital, Osaka 556-0017, Osaka, Japan; 3 Department of Internal Medicine (IV), Division of Rheumatology, Osaka Medical and Pharmaceutical University, Takatsuki 569-8686, Osaka, Japan; shogo.matsuda@ompu.ac.jp (S.M.); aya.sakamoto@ompu.ac.jp (A.S.); yoshihiro.shou@ompu.ac.jp (Y.S.); tooru.takeuchi@ompu.ac.jp (T.T.); 4 Department of Radiology, Ikeda City Hospital, Ikeda 563-8510, Osaka, Japan

**Keywords:** dermatomyositis, polymyositis, interstitial lung disease, progressive pulmonary fibrosis, anti-melanoma differentiation-associated gene 5 antibody, anti-aminoacyl tRNA synthetase antibody

## Abstract

**Background/Objectives**: This study compared chest high-resolution computed tomography (HRCT) findings between patients with anti-melanoma differentiation-associated gene 5 (MDA5) antibody-positive and antibody-negative progressive pulmonary fibrosis (PPF) with polymyositis/dermatomyositis (PM/DM). **Methods**: Of the 85 patients with PM/DM-interstitial lung disease (ILD), 17 were anti-MDA5 antibody-positive, and 68 were antibody-negative. Among these, 5 anti-MDA5 antibody-positive and 9 antibody-negative cases met the criteria for PPF and were enrolled in the study. The chest HRCT findings and the duration from treatment initiation to the appearance of key fibrotic changes were analyzed. **Results**: In the anti-MDA5-positive group, all patients were diagnosed with PPF within 6 months of treatment initiation, compared to only 22.2% in the anti-MDA5-negative group. While there was no difference between the anti-MDA5 antibody-positive and antibody-negative groups in terms of chest HRCT findings associated with PPF, the duration to the appearance of increased traction bronchiectasis and bronchiolectasis, and new ground-glass opacity with traction bronchiectasis was significantly shorter in the anti-MDA5-positive group (*p* = 0.016 and *p* = 0.023, respectively). The appearance of new fine reticulations and increased coarseness of reticular abnormalities tended to be shorter in the anti-MDA5 antibody-positive group than in the antibody-negative group. **Conclusions**: Pulmonary fibrosis in patients with anti-MDA5 antibody-positive ILD can rapidly progress within 6 months, despite immunosuppressive therapy. Frequent HRCT monitoring and early combination therapy with antifibrotic agents are crucial for managing the progression of fibrosis.

## 1. Introduction

Polymyositis (PM) and dermatomyositis (DM) are classified as idiopathic inflammatory myopathies (IIMs), characterized by inflammation that can involve multiple organ systems, including the skin, lungs, and cardiovascular tissues [1,2]. Interstitial lung disease (ILD) frequently complicates PM/DM, which is a life-threatening condition [3]. The major autoantibodies associated with PM/DM-ILD include the anti-aminoacyl tRNA synthetase (ARS) antibodies and anti-melanoma differentiation-associated gene 5 (MDA5) antibodies [4,5]. In cases where ILD progresses with positive anti-ARS antibodies, immunosuppressive therapies with glucocorticoids (GCs), calcineurin inhibitors, and/or intermittent intravenous cyclophosphamide (IVCY) are administered and are usually effective, although refractory and recurrent cases pose significant challenges [6,7]. Conversely, patients with positive anti-MDA5 antibodies require early and aggressive combination therapy with GCs, calcineurin inhibitors, and IVCY; however, many patients still succumb to respiratory failure within a few months despite treatments [8].

Progressive fibrosing ILD (PF-ILD) refers to a subset of ILDs characterized by progressive fibrosis regardless of the underlying cause [9,10] and is defined as worsening of respiratory symptoms, declining lung function, and increased fibrosis observed on high-resolution computed tomography (HRCT). This category includes connective tissue disease-associated ILDs. The broader term, progressive pulmonary fibrosis (PPF), has been proposed [11] and encompasses all ILDs with progressive fibrosis, including idiopathic pulmonary fibrosis. PF-ILD and PPF play important roles in the selection of patients for nintedanib and other antifibrotic therapies that are currently in development [10,12].

Despite early treatment initiation, lung fibrosis often progresses in PM/DM-ILD, leading to a poor prognosis. Zanatta et al. observed that 18% of IIM-ILD cases progressed to PF-ILD within 1 year, with univariate predictors of PF-ILD including positive anti-MDA5 antibodies, heliotrope rash, xerostomia, and dry eyes [13]. Wang et al. found that 30.6% of patients with myositis-specific autoantibodies developed PPF during follow-up, with risk factors including acute/subacute (A/S) ILD onset, low predicted diffusion capacity of the lungs for carbon monoxide (DLCO)%, and diffuse alveolar damage patterns on chest HRCT [14]. However, the knowledge of PPF in PM/DM remains limited. Detailed chest HRCT findings in PM/DM-PPF cases, especially regarding the differences between associated autoantibodies, remain unclear. Therefore, this study aimed to examine and compare detailed chest HRCT findings between patients with anti-MDA5 antibody-positive and antibody-negative PM/DM-PPF.

## 2. Materials and Methods

### 2.1. Patients

We reviewed patients treated at the Osaka Medical and Pharmaceutical University Hospital from April 2015 to March 2022. PM, DM, or clinically amyopathic DM was classified based on the Bohan and Peter criteria [1,2] or the Sontheimer and Gerami criteria [15,16]. When the classification criteria were not met, antisynthetase syndrome was considered in the presence of an anti-ARS antibody and the occurrence of at least one of the following conditions: arthritis, myositis, and ILD [17]. ILD detection was performed using chest HRCT. ILD was classified as A/S-ILD or chronic ILD. A/S-ILD was defined as rapid worsening of the respiratory condition, laboratory findings, arterial blood gas findings, chest HRCT images, and pulmonary function test findings within 3 months of onset [18]. Chronic ILD was defined as cases that did not meet the criteria for A/S-ILD. Disease duration was determined as the time from the onset of ILD-related respiratory symptoms to hospital admission. Clinical data were collected from medical records. This study adhered to the principles of the Declaration of Helsinki and its amendments and was approved by the Ethics Committee of Osaka Medical and Pharmaceutical University (approval nos. 1529 and 1598). Informed consent was obtained from all participants.

### 2.2. Measurement of Laboratory Parameters

Baseline serum creatine kinase, aldolase, lactic acid dehydrogenase, C-reactive protein, Krebs von den Lungen-6, ferritin, and pulmonary surfactant protein (Sp-D) were measured. Anti-ARS antibodies were analyzed using an enzyme-linked immunosorbent assay (ELISA; MESACUP; MBL, Nagoya, Japan) and a blot assay (Myositis Profile Euroline Blot test kit; EUROIMMUN, Lübeck, Germany). Anti-MDA5 antibodies were examined using ELISA (MESACUP; MBL, Nagoya, Japan). The cutoff value for negativity was set at an index of less than 5, according to the manufacturer’s instructions. The antibody index was assessed before treatment initiation and at 3 and 6 months after treatment initiation, where available. The rate of decrease in the anti-MDA5 antibody index was calculated by dividing the post-treatment index by the pre-treatment index.

### 2.3. Assessment of Respiratory Symptoms, Arterial Blood Gas Analysis, and Pulmonary Function Testing (PFT)

Respiratory symptoms were assessed using the modified Medical Research Council grades, which is a five-point scale based on the severity of breathlessness related to physical activity [19]. The alveolar-arterial oxygen gradient (AaDO_2_) was calculated using blood gas analysis at admission as previously described [20]. During PFTs, static and dynamic lung volumes were measured using spirometry (SYSTEM21; Minato Medical Science, Osaka, Japan). Forced vital capacity (FVC) was assessed using the N_2_ washout method, and DLCO was evaluated using the single-breath method. The PFT results were expressed as a percentage of the predicted values.

### 2.4. Interpretation of Chest HRCT Findings

HRCT was performed using a 64-detector row computed tomography Aquilon multiscanner (Canon Medical Systems Corporation, Tokyo, Japan). The slice thickness was set to 1.0 mm, and the scan covered the entire lung field. Chest HRCT images were obtained from all patients before treatment initiation and as needed during induction therapy for remission. Chest HRCT was performed at least once a year to monitor ILD progression, even during the maintenance phase of remission. Images were reviewed independently by two radiologists specializing in the interpretation of chest images (NS and MK), who were blinded to the patients’ clinical information. Disagreements between observers were resolved through consensus.

### 2.5. Definition of PPF

PPF was defined as at least 2 of the 3 criteria (worsening respiratory symptoms, radiological progression, and physiological progression) occurring within the past year, with no alternative explanation in a patient with ILD as previously defined [11]. Physiological evidence of disease progression was defined as either an absolute decline in FVC of ≥5% within 1 year of follow-up or an absolute decline in DLCO, which was corrected for hemoglobin, of ≥10% within 1 year of follow-up. Radiological evidence of disease progression was defined as at least one of the following: increased extent or severity of traction bronchiectasis and bronchiolectasis; new ground-glass opacity (GGO) with traction bronchiectasis; new fine reticulations; increased extent or coarseness of reticular abnormalities; new or increased honeycombing; and increased lobar volume loss.

### 2.6. Treatments

GCs were administered to all patients who received treatment, with prednisolone being used in all cases. The dosage of prednisolone (mg/day) was determined by the attending physician according to the patient’s condition. Cyclosporin-A (CSA) and tacrolimus (TAC) were administered as a combination therapy at the physician’s discretion. CSA was started at 4 mg/kg/day as a single dose before breakfast, with the 2-h post-administration concentration adjusted to ≥1500 ng/mL. TAC was initiated at 0.075 mg/kg/day in two divided doses before breakfast and dinner, with the trough concentration adjusted to 5–10 ng/mL in anti-MDA5 antibody-negative patients. In anti-MDA5 antibody-positive patients, the trough concentration of TAC was maintained at 10–15 ng/mL for the first 3 months following treatment initiation and subsequently maintained at 5–10 ng/mL. IVCY was initiated at 750–1000 mg/m^2^ body surface area. In patients positive for anti-MDA5 antibody, the initial treatment consisted of a simultaneous administration of triple therapy: GCs, CSA or TAC, and IVCY. Additional treatments, including methylprednisolone (MPDN) pulse therapy, intravenous immunoglobulin, plasma exchange (PE), mycophenolate mofetil, rituximab, and tofacitinib, were provided based on each patient’s condition at the physician’s discretion.

### 2.7. Statistical Analysis

Data are presented as median (interquartile range). Fisher’s exact test was used when appropriate, and the Mann–Whitney U test was used to compare median values. Statistical significance was set at *p* < 0.05. Data were analyzed using JMP version 17.0 (SAS Institute Inc., Cary, NC, USA).

## 3. Results

### 3.1. Comparison of the Baseline Clinical Characteristics of PPF with Anti-MDA5 Antibody-Positive and Antibody-Negative Cases

**Figure 1:**
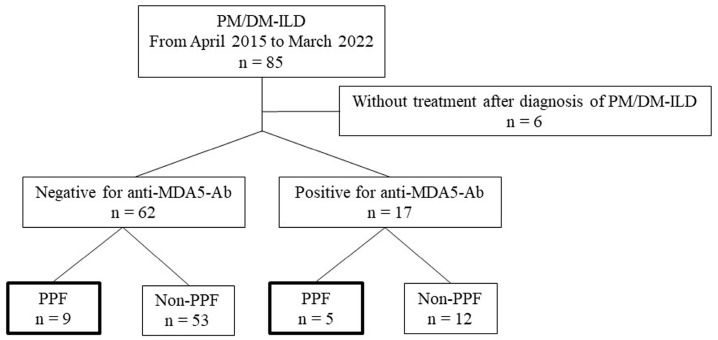
Flowchart depicting the classification of patients with PM/DM-ILD into anti-MDA5 antibody-positive and antibody-negative groups, and the selection of patients meeting the definition of PPF. Between April 2015 and March 2022, 85 patients with PM/DM-ILD were treated at our institution, including 17 anti-MDA5 antibody-positive and 68 antibody-negative patients. Six cases without therapeutic intervention were excluded from the analysis. Among these, 5 of 17 antibody-positive patients and 9 of 62 antibody-negative patients met the PPF criteria. PM/DM, polymyositis/dermatomyositis; ILD, interstitial lung disease; MDA5, melanoma differentiation-associated gene 5; Ab, antibody; PPF, progressive pulmonary fibrosis.

Figure 1 presents a flowchart depicting the classification of patients with PM/DM-ILD into anti-MDA5 antibody-positive and antibody-negative groups, and the extraction of patients meeting the definition of PPF. Between April 2015 and March 2022, 85 patients with PM/DM-ILD were treated at our institution, of whom 17 tested positive for anti-MDA5 antibodies and 68 tested negative. Six cases (all anti-MDA5 antibody-negative) without therapeutic intervention were excluded from the analysis. The baseline clinical characteristics of patients from 17 anti-MDA5 antibody-positive and 62 antibody-negative cases are presented in Table 1. Out of the 50 cases that received positive test results for anti-ARS antibodies by ELISA, a blot assay was additionally performed in 48 cases, revealing positivity for anti-Jo-1 antibodies in 15 cases, anti-EJ antibodies in 17 cases, anti-PL7 antibodies in 10 cases, and anti-PL12 antibodies in 2 cases. The antibody-positive group had a significantly higher proportion of A/S-ILD, shorter disease duration and follow-up duration, elevated serum Sp-D and ferritin levels, and higher AaDO_2_ (*p* = 0.0001, <0.0001, 0.04, <0.0001, <0.0001, and 0.001, respectively) than did the antibody-negative group. Additionally, the antibody-positive group received significantly higher doses of GC and had a higher rate of additional therapies. The baseline clinical characteristics of 14 PPF cases and 65 non-PPF cases are shown in Table 2. The PPF group had significantly higher baseline AaDO_2_ and significantly lower %FVC and %DLCO than did the non-PPF group (*p* = 0.03, 0.01, and 0.03, respectively). There was no significant difference in the pretreatment anti-MDA5 antibody index between the PPF and non-PPF groups. At 3 months after treatment initiation, the antibody index was available in 3 cases in the PPF group and 10 cases in the non-PPF group. At 6 months, the index was available in 3 cases in the PPF group and 6 cases in the non-PPF group. One patient in the non-PPF group showed seroconversion to negativity during follow-up. The rate of decrease in the anti-MDA5 antibody index at 3 and 6 months did not differ significantly between the PPF and non-PPF groups. Among the study cases, one patient in the antibody-negative group was diagnosed with lung adenocarcinoma at the time of treatment initiation, and another patient in the anti-ARS antibody-positive group was diagnosed with lung adenocarcinoma 1 year after treatment initiation. Neither of these patients met the criteria for PPF.

Among the anti-MDA5 antibody-positive and antibody-negative cases, 5 (29.4%) and 9 (14.5%) cases, respectively, met the criteria for PPF (Table 3). The median disease duration was significantly longer in the anti-MDA5 antibody-negative group (19.6 weeks) than in the anti-MDA5 antibody-positive group (0.29 weeks; *p* = 0.003). Similarly, the median follow-up duration was significantly longer in the anti-MDA5 antibody-negative group (3.3 years) than in the anti-MDA5 antibody-positive group (0.1 years; *p* = 0.011). The median serum Sp-D levels were notably higher in the anti-MDA5 antibody-negative group (246 ng/mL) than in the anti-MDA5 antibody-positive group (48.3 ng/mL; *p* = 0.012). Conversely, the median serum levels of ferritin and AaDO_2_ were higher in the anti-MDA5 antibody-positive group (1085 ng/mL and 85.3 torr, respectively) compared with those in the anti-MDA5 antibody-negative group (225.2 ng/mL and 21.6 torr, respectively; *p* = 0.016 and *p* = 0.008, respectively). The median initial GC dose was significantly higher in the anti-MDA5 antibody-positive group than in the anti-MDA5 antibody-negative group (*p* = 0.044). The utilization rates of MPDN pulse therapy and PE were higher in the anti-MDA5 antibody-positive group than in the anti-MDA5 antibody-negative group (*p* = 0.003 and *p* = 0.023, respectively). In the anti-MDA5 antibody-positive PPF group, 2 patients, and in the antibody-negative PPF group, 1 patient, died of respiratory failure due to ILD progression. Notably, all these deaths occurred after the criteria for PPF were met.

### 3.2. Timing of New Diagnosis with PPF After Treatment Initiation in Patients with PM/DM-ILD

The timing of new PPF diagnoses following treatment initiation in patients with PM/DM-ILD is outlined in Table 4. In the anti-MDA5 antibody-positive group, all 5 patients were diagnosed with PPF within 6 months of treatment initiation. Conversely, among the 9 patients in the anti-MDA5 antibody-negative group, only 2 (22.2%) were diagnosed with PPF within 6 months of treatment initiation. Five (55.6%) patients were diagnosed with PPF more than 2 years after treatment initiation, and there were 2 patients where PPF was diagnosed more than 5 years after treatment initiation. Table S1 shows the 2-year changes in %DLCO after treatment initiation in PPF cases. Among anti-MDA5-Ab positive cases, a rapid decline in %DLCO was observed within the first 6 months of treatment. In contrast, anti-MDA5-Ab negative cases exhibited a slower decline or gradual improvement over time.

### 3.3. Comparison of Chest HRCT Findings Regarding PPF Between Anti-MDA5 Antibody Positive and Negative Groups

**Figure 2:**
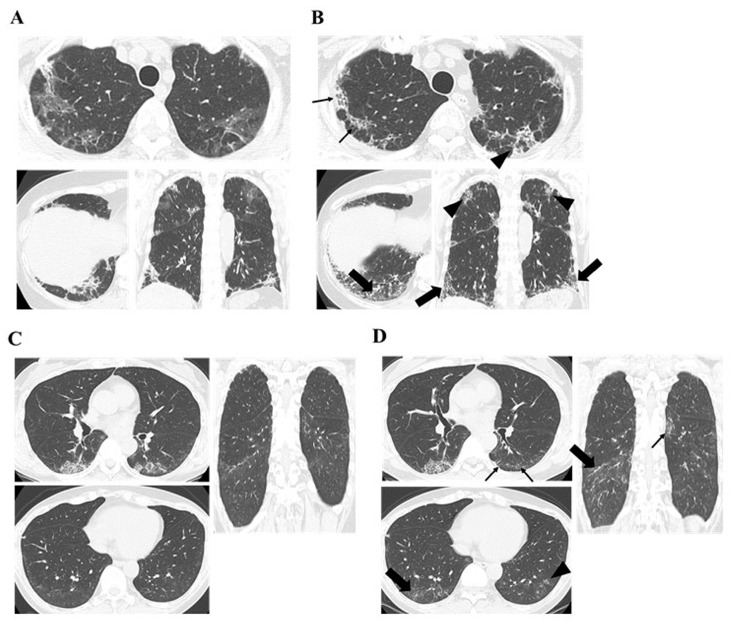
Chest HRCT findings regarding PPF between anti-MDA5 antibody-positive and antibody-negative patients. Progressive appearance or expansion of fibrotic changes defined as PPF was observed. (**A**,**B**) Anti-MDA5 antibody-positive PPF case, where (**A**) was before treatment and (**B**) was 4 months after treatment initiation. The large arrows indicate an increased extent or severity of traction bronchiectasis and bronchiolectasis, and new ground-glass opacity with traction bronchiectasis. The small arrows indicate an increased extent or increased coarseness of reticular abnormality. The arrowheads indicate new fine reticulation. (**C**,**D**) Anti-MDA5 antibody-negative (anti-ARS antibody-positive) PPF case, where (**C**) was before treatment and (**D**) was 7 months after treatment initiation. The large arrows indicate new ground-glass opacity with traction bronchiectasis. The small arrows indicate increased extent or increased coarseness of reticular abnormality. The arrowheads indicate new fine reticulation. HRCT, high-resolution computed tomography; PPF, progressive pulmonary fibrosis; MDA5, melanoma differentiation-associated gene 5. ARS, aminoacyl tRNA synthetase.

A comparison of chest HRCT findings regarding PPF between antibody-positive and antibody-negative cases for anti-MDA5 is presented in Table 5. The duration from treatment initiation to the appearance of increased extent or severity of traction bronchiectasis and bronchiolectasis, and new GGO with traction bronchiectasis, was significantly shorter in the anti-MDA5 antibody-positive group than in the anti-MDA5 antibody-negative group (*p* = 0.016 and *p* = 0.023, respectively). In addition, the time from treatment initiation to the appearance of new fine reticulations and increased extent or coarseness of reticular abnormality tended to be shorter in the anti-MDA5 antibody-positive group than in the anti-MDA5 antibody-negative group (*p* = 0.16 and *p* = 0.067, respectively). The incidence of chest HRCT findings associated with PPF after treatment initiation did not differ between the anti-MDA5 antibody-positive and antibody-negative groups. Figure 2 shows the progression of chest HRCT findings in the anti-MDA5 antibody-positive and antibody-negative cases that progressed to PPF. Additionally, a comparative analysis was conducted between the anti-MDA5 antibody-positive ILD group (all cases being A/S-ILD) and the anti-MDA5 antibody-negative A/S-ILD cases, yielding similar results (Table S2).

## 4. Discussion

The results from the detailed evaluation of chest HRCT highlight the differences in the frequency and timing of PPF-related findings between anti-MDA5 antibody-positive and antibody-negative PM/DM-ILD patients. All anti-MDA5 antibody-positive patients met the PPF criteria within 6 months of treatment initiation, while only 2 patients in the antibody-negative group met the criteria. Progression to PPF in antibody-negative cases occurred over a longer period. Although the types of HRCT findings associated with PPF were similar between the two groups, the time to new-onset or exacerbation of traction bronchiectasis, bronchiolectasis, and traction bronchiectasis with GGO or reticulation was significantly shorter in the antibody-positive group.

Anti-MDA5 antibody-positive ILD is characterized by an A/S course, rapid progression, and severe respiratory distress [21]. HRCT findings frequently include GGO (75.7%), consolidation (64.4%), and reticulation (55.3%), often accompanied by traction bronchiectasis, with honeycombing being rare [22]. These findings are predominantly subpleural and peribronchovascular [23,24,25]. GGO and consolidation strongly indicate disease progression, often with rapid increases in traction bronchiectasis during exacerbations [21,26,27]. Despite treatment, fibrosis progression is frequently irreversible [28]. In this study, all anti-MDA5 antibody-positive PPF cases met the PPF criteria within 6 months of treatment initiation. HRCT findings included new GGO with traction bronchiectasis and progression of traction bronchiectasis and bronchiolectasis in all cases. Fine reticulations (60%) and coarsened reticular abnormalities with lobar volume loss (40%) were also observed. This study highlights the characteristic expansion of fibrosis, that is, traction bronchiectasis, bronchiolectasis, reticulation, and lobar volume loss without honeycombing, emphasizing the importance of frequent HRCT monitoring for early detection and timely management of fibrosis progression.

Anti-ARS antibody-positive ILD typically shows chronic to subacute progression and responds well to initial treatment, though relapses are common [6]. HRCT findings often include GGO, consolidations, and reticulations distributed around the bronchovascular bundles and lower lobe periphery, frequently accompanied by traction bronchiectasis [29]. With disease progression, these features expand along with lobar volume loss, despite a good initial response to immunosuppressive therapy [29,30]. In this study, 77.8% of anti-MDA5 antibody-negative cases were anti-ARS antibody-positive. Among these, over half required more than 2 years to meet the PPF criteria. HRCT findings included new GGO with traction bronchiectasis, progression of traction bronchiectasis and bronchiolectasis, and the appearance of fine reticulations and coarsened reticular abnormalities, with minimal honeycombing or lobar volume loss. This study highlights the frequent expansion of traction bronchiectasis, bronchiolectasis, and reticulation in anti-ARS antibody-positive PPF, while new honeycombing and lobar volume loss remain infrequent. These findings provide new insights into the radiological progression and clinical characteristics of the disease.

The time to meet PPF criteria was significantly shorter in anti-MDA5 antibody-positive cases due to the rapid progression of ILD. However, no differences in fibrotic findings on HRCT were observed between antibody-positive and antibody-negative PPF cases. Although GGO and consolidation markers of disease activity [26,27] differ between these groups, fibrotic patterns on HRCT appear similar.

This study demonstrated that pulmonary fibrosis in anti-MDA5 antibody-positive ILD can progress rapidly within 6 months, even after initiating immunosuppressive therapy. All anti-MDA5 antibody-positive PPF cases showed HRCT findings defined by the PPF criteria, including increased traction bronchiectasis and bronchiolectasis and new GGO with traction bronchiectasis. Frequent HRCT monitoring may enable early detection of fibrosis progression, and early combination therapy with antifibrotic agents like nintedanib alongside intensified immunosuppressive therapy could help prevent progression. In anti-MDA5 antibody-negative ILD, longitudinal HRCT imaging to track the progression of traction bronchiectasis and bronchiolectasis, new GGO with traction bronchiectasis, fine reticulation, and coarsened reticular abnormalities may indicate the timing of antifibrotic therapy to effectively manage fibrosis progression.

Although the patients diagnosed with lung adenocarcinoma in this study did not meet the criteria for PPF, these cases highlight the need for careful monitoring of lung cancer in patients with ILD. Given that radiation therapy can potentially exacerbate ILD, the decision to initiate radiation therapy should be made with caution, considering the potential impact on disease progression.

This study has several limitations. First, the small sample size, particularly in the anti-MDA5 antibody-positive group, limits the generalizability of the findings and reduces statistical power. Second, the retrospective and single-center design introduces potential biases, including those related to the diagnostic and treatment protocols. Third, differences in follow-up duration between groups, with a median of 3.3 years in the anti-MDA5 antibody-negative group compared to 1.0 year in the positive group, may have influenced the PPF diagnosis timing. Additionally, the higher frequency of chest HRCT scans during the early post-treatment phase in the antibody-positive group could have introduced a bias. Prospective multicenter studies with standardized HRCT intervals are needed to confirm these findings, optimize management strategies, and assess the ability of pre-treatment chest HRCT findings to predict progression to PPF, which would be highly valuable for early intervention and improved patient outcomes.

## 5. Conclusions

Although differences in PPF-related HRCT findings were not observed between the anti-MDA5 antibody-positive and antibody-negative groups, pulmonary fibrosis in anti-MDA5 antibody-positive ILD progressed rapidly within six months, despite immunosuppressive therapy. Frequent monitoring of HRCT findings, such as increased traction bronchiectasis and new GGO with traction bronchiectasis, is essential for early detection and timely intervention. Early combination therapy with antifibrotic agents may help prevent progression. In anti-MDA5 antibody-negative ILD, longitudinal HRCT is necessary to guide the initiation of antifibrotic therapy.

## Figures and Tables

**Table 1:** Comparison of baseline clinical characteristics between anti-MDA5-Ab positive and negative cases.

Characteristics	Anti-MDA5 Negative (*n* = 62)	Anti-MDA5 Positive (*n* = 17)	*p*-Value
Age, years	60.7 (51.4–68.5)	58.4 (53.5–66.8)	0.61
Females, n (%)	45 (73)	12 (71)	1
CADM/DM/PM/ASSD, n (%)	15 (24.2)/14 (22.6)/2 (3.2)/31 (50.0)	9 (52.9)/8 (47.1)/0 (0)/0 (0)	
A/S-ILD, n (%)	32 (51.6)	17 (100)	0.0001
Anti-MDA5-Ab, index		2350 (150–3800) ^l^	
Anti-ARS-Ab (+), DN, n (%)	50 (80.7), 12 (19.4)		
Jo-1/EJ/PL7/PL12/others	15/17/10/2/2		
Anti-Mi-2-Ab (+), n (%)	2 (3.2)		
Anti-TIF1-γ-Ab (+), n (%)	1 (1.6)		
Disease duration, weeks	7.9 (3.9–23.9)	0.16 (0–1.4)	<0.0001
Follow-up duration, years	3.3 (0.9–4.6)	1.0 (0.1–4.1)	0.04
Modified MRC scale	1 (0–2)	1 (1–2)	0.24
**Laboratory findings**			
CK, U/L	107 (69.8–435.8)	268 (111.5–622.5)	0.24
ALD, U/L	6.3 (4.1–16.3)	8.6 (6.9–10.4)	0.28
CRP, mg/mL	0.3 (0.06–0.83)	0.6 (0.18–2.08)	0.14
KL-6, U/mL	1002.5 (569–1432.8)	789 (374–1180.5)	0.2
Sp-D, ng/mL	167 (106–277) ^a^	43.3 (27.5–70.4) ^b^	<0.0001
Ferritin, ng/mL	176.5 (78.8–350.8) ^c^	1054 (536.5–1817.5) ^d^	<0.0001
AaDO_2_	19.0 (9.0–26.5) ^e^	35.1 (22.7–68.9) ^d^	0.001
**PFT findings**			
Predicted FVC, %	76.8 (64.8–88) ^f^	76 (67.1–90.6) ^g^	0.8
Predicted DLCO, %	46.0 (33.5–58.5) ^h^	40.8 (36.8–48.4) ^i^	0.57
**Treatment**			
GCs, mg/day	47.5 (33.8–55)	55 (50–65)	0.01
CSA, mg/day	250 (200–275) ^j^	287.5 (243.8–306.3) ^b^	0.89
TAC, mg/day	6 (3–8) ^f^	1 (0–6) ^k^	0.03
MPDN pulse, n (%)	8 (13.1)	11 (64.7)	<0.0001
IVCY, n (%)	30 (48.4)	17 (100)	<0.0001
IVIG, n (%)	3 (5.3)	8 (47.1)	0.0002
PE, n (%)	4 (6.6)	12 (70.6)	<0.0001
MMF, n (%)	5 (8.1)	5 (29.4)	0.03
RTX, n (%)	1 (1.6)	4 (23.5)	0.01
TOF, n (%)	1 (1.6)	9 (52.9)	<0.0001
Nintedanib, n (%)	6 (9.7)	1 (5.9)	1
**Outcomes**			
Dead due to ILD, n (%)	4 (6.6)	4 (25)	0.053

The laboratory markers are presented as median (interquartile range). The *p*-values were estimated using Fisher’s exact test or Mann-Whitney U test. ^a^ n = 47; ^b^ n = 14; ^c^ n = 58; ^d^ n = 17; ^e^ n = 56; ^f^ n = 51; ^g^ n = 5; ^h^ n = 50; ^i^ n = 4; ^j^ n = 19; ^k^ n = 13, ^l^ n = 15. MDA5, melanoma differentiation-associated gene 5; Ab, antibody; CADM, clinically amyopathic dermatomyositis; DM, dermatomyositis; PM, polymyositis; ASSD, anti-synthetase syndrome; A/S, acute/subacute; ILD, interstitial lung disease; ARS, Aminoacyl-tRNA synthetase; TIF1-γ, transcriptional intermediary factor 1-γ; MRC, Medical Research Council; CK, creatine kinase; ALD, aldolase; CRP, C-reactive protein; KL-6, Krebs von den Lungen-6; Sp-D, surfactant protein-D; AaDO_2_, alveolar-arterial oxygen difference; PFT, pulmonary function test; FVC, forced vital capacity; DLCO, diffusion capacity of the lung for carbon monoxide; GCs, glucocorticoids; CSA, cyclosporin-A; TAC, tacrolimus; MPDN, methylprednisolone; IVCY, intravenous cyclophosphamide; IVIG, intravenous immunoglobulin; PE, plasma exchange; MMF, mycophenolate mofetil; RTX, rituximab; TOF, tofacitinib.

**Table 2:** Comparison of baseline clinical characteristics between PPF and non PPF cases.

Characteristics	Non PPF (*n* = 65)	PPF (*n* = 14)	*p*-Value
Age, years	60.3 (52.2–68.3)	61.9 (51.1–67.8)	0.96
Females, n (%)	46 (71)	11 (79)	0.75
CADM/DM/PM/ASSD, n (%)	17 (26.2)/20 (30.8)/1 (1.5)/27 (41.5)	7 (50.0)/2 (14.3)/1 (7.1)/4 (28.6)	
A/S-ILD, n (%)	38 (58.5)	11 (78.6)	0.23
Anti-MDA5-Ab (+), Anti-ARS-Ab (+), DN, n (%)	12 (18.5), 44 (67.7), 9 (13.9)	5 (35.7), 6 (42.9), 3 (21.4)	
Anti-MDA5-Ab, index	1450 (150–3250) ^m^	3675 (862.5-5287.5) ^n^	0.21
Anti-Mi-2-Ab (+), n (%)	2 (3.2)	0 (0)	1
Anti-TIF1-γ-Ab (+), n (%)	1 (1.6)	0 (0)	1
Disease duration, weeks	7.0 (1.6–17.0)	3.8 (0.5–21.5)	0.77
Follow-up duration, years	2.9 (0.9–4.6)	1.2 (0.1–3.5)	0.06
Modified MRC scale	1 (0–2)	1.5 (1–3)	0.19
**Laboratory findings**			
CK, U/L	123 (70.5–435.5)	268 (89.8–717.5)	0.12
ALD, U/L	7.1 (4.6–14.7)	7.6 (4.7–13.0)	0.63
CRP, mg/mL	0.25 (0.09–1.70)	0.6 (0.27–1.54)	0.18
KL-6, U/mL	967 (512.5–1272.5)	1162 (734–1866.6)	0.15
Sp-D, ng/mL	147.5 (68.9–267) ^a^	116.5 (44.4–293) ^b^	0.61
Ferritin, ng/mL	204.7 (96.3–609.8) ^c^	382 (134.6–1340) ^d^	0.14
AaDO_2_	19.8 (10.1–29.3) ^e^	31.4 (19.4–79.1) ^f^	0.03
**PFT findings**			
Predicted FVC, %	78.3 (66.1–89.2) ^g^	63.1 (59.8–69.5) ^b^	0.01
Predicted DLCO, %	47.4 (36.3–60.9) ^h^	35.7 (25.4–47.3) ^i^	0.03
**Treatment**			
GCs, mg/day	50 (35–55)	57.5 (33.8–66.3)	0.19
CSA, mg/day	250 (218.8–300) ^j^	250 (250–300) ^k^	0.89
TAC, mg/day	5.5 (2.8–7.3) ^l^	6 (3–7) ^b^	0.81
MPDN pulse, n (%)	13 (20.3)	6 (42.9)	0.09
IVCY, n (%)	36 (55.4)	11 (78.6)	0.14
IVIG, n (%)	7 (11.3)	4 (33.3)	0.07
PE, n (%)	11 (17.2)	5 (35.7)	0.15
MMF, n (%)	8 (12.3)	2 (14.3)	1
RTX, n (%)	4 (6.2)	1 (7.1)	1
TOF, n (%)	7 (10.9)	3 (21.4)	0.37
Nintedanib, n (%)	5 (7.7)	2 (14.3)	0.6
**Outcomes**			
Dead due to ILD, n (%)	5 (7.8)	3 (23.1)	0.13

The laboratory markers are presented as median (interquartile range). The *p*-values were estimated using Fisher’s exact test or Mann-Whitney U test. ^a^ n = 51; ^b^ n = 10; ^c^ n = 62; ^d^ n = 13; ^e^ n = 59; ^f^ n = 14; ^g^ n = 46; ^h^ n = 45, ^i^ n = 9, ^j^ n = 26, ^k^ n = 7, ^l^ n = 54, ^m^ n = 11, ^n^ n = 4. PPF, progressive pulmonary fibrosis; MDA5, melanoma differentiation-associated gene 5; Ab, antibody; CADM, clinically amyopathic dermatomyositis; DM, dermatomyositis; PM, polymyositis; ASSD, anti-synthetase syndrome; A/S, acute/subacute; ILD, interstitial lung disease; ARS, Aminoacyl-tRNA synthetase; TIF1-γ, transcriptional intermediary factor 1-γ; MRC, Medical Research Council; CK, creatine kinase; ALD, aldolase; CRP, C-reactive protein; KL-6, Krebs von den Lungen-6; Sp-D, surfactant protein-D; AaDO_2_, alveolar-arterial oxygen difference; PFT, pulmonary function test; FVC, forced vital capacity; DLCO, diffusion capacity of the lung for carbon monoxide; GCs, glucocorticoids; CSA, cyclosporin-A; TAC, tacrolimus; MPDN, methylprednisolone; IVCY, intravenous cyclophosphamide; IVIG, intravenous immunoglobulin; PE, plasma exchange; MMF, mycophenolate mofetil; RTX, rituximab; TOF, tofacitinib.

**Table 3:** Comparison of baseline clinical characteristics of PPF in anti-MDA5-Ab positive and negative cases.

Characteristics	Anti-MDA5 Negative (*n* = 9)	Anti-MDA5 Positive (*n* = 5)	*p*-Value
Age, years	58 (47.3–68.7)	65.9 (60.9–67.7)	0.42
Females, n (%)	8 (89)	3 (60)	0.51
CADM/DM/PM/ASSD, n (%)	3 (33.3)/1 (11.1)/1 (11.1)/4 (44.4)	4 (80.0)/1 (20.0)/0 (0)/0 (0)	
A/S-ILD, n (%)	6 (66.7)	5 (100)	0.26
Anti-MDA5-Ab, index		3675 (862.5-5287.5) ^c^	
Anti-ARS-Ab positive, DN, n (%)	7 (70), 3 (30)		
Anti-Mi-2-Ab (+), n (%)	0 (0)		
Anti-TIF1-γ-Ab (+), n (%)	0 (0)		
Disease duration, weeks	19.6 (3.9–29.4)	0.29 (0–1.4)	0.003
Follow-up duration, years	3.3 (1.0–4.6)	0.1 (0.1–0.7)	0.011
Modified MRC scale	1 (0.5–2.5)	2 (1–3.5)	0.41
**Laboratory findings**			
CK, U/L	300 (107.5–1089)	236 (74–693.5)	0.69
ALD, U/L	9 (3.8–30.4)	6.8 (5.75–8.85)	0.59
CRP, mg/mL	0.5 (0.08–0.96)	1.49 (0.89–2.56)	0.07
KL-6, U/mL	1376 (934.5–1876.1)	771 (533.5–1700)	0.23
Sp-D, ng/mL	246 (174–489.5) ^a^	48.3 (24.2–71.2)	0.012
Ferritin, ng/mL	225.2 (96.275–36.5) ^b^	1085 (638.5–2152.5)	0.016
AaDO_2_	21.6 (16.6–31.4)	85.3 (36.9–103.5)	0.008
**PFT findings**			
Predicted FVC, %	61.9 (59.5–65.3) ^b^	78.6 (62.3–94.9) ^c^	0.36
Predicted DLCO, %	32.4 (23.2–44.9) ^d^	43.2 (36.7–49.6) ^c^	0.31
**Treatment**			
GCs (n = 14), mg/kg	50 (30–60)	60 (60–75)	0.044
CSA (n = 7), mg/day	250 (100–287.5) ^e^	300 (250–300) ^f^	0.34
TAC (n = 10), mg/day	6 (4–6.3) ^g^	3.5 (0–10.8) ^e^	1
MPDN pulse, n (%)	1 (11.1)	5 (100)	0.003
IVCY, n (%)	6 (66.7)	5 (100)	0.26
IVIG, n (%)	1 (14.3)	3 (60)	0.22
PE, n (%)	1 (11.1)	4 (80)	0.023
MMF, n (%)	1 (11.1)	1 (20)	1
RTX, n (%)	0 (0)	1 (20)	0.36
TOF, n (%)	1 (11.1)	2 (40)	0.51
Nintedanib, n (%)	1 (11.1)	1 (20)	1
**Outcomes**			
Dead due to ILD, n (%)	1 (11.1)	2 (40)	0.51

The laboratory markers are presented as median (interquartile range). The *p*-values were estimated using Fisher’s exact test or Mann-Whitney U test. ^a^ n = 5; ^b^ n = 8; ^c^ n = 2; ^d^ n = 7; ^e^ n = 4; ^f^ n = 3; ^g^ n = 6. PPF, progressive pulmonary fibrosis; MDA5, melanoma differentiation-associated gene 5; Ab, antibody; CADM, clinically amyopathic dermatomyositis; DM, dermatomyositis; PM, polymyositis; ASSD, anti-synthetase syndrome; A/S, acute/subacute; ILD, interstitial lung disease; ARS, Aminoacyl-tRNA synthetase; TIF1-γ, transcriptional intermediary factor 1-γ; MRC, Medical Research Council; CK, creatine kinase; ALD, aldolase; CRP, C-reactive protein; KL-6, Krebs von den Lungen-6; Sp-D, surfactant protein-D; AaDO_2_, alveolar-arterial oxygen difference; PFT, pulmonary function test; FVC, forced vital capacity; DLCO, diffusion capacity of the lung for carbon monoxide; GCs, glucocorticoids; CSA, cyclosporin-A; TAC, tacrolimus; MPDN, methylprednisolone; IVCY, intravenous cyclophosphamide; IVIG, intravenous immunoglobulin; PE, plasma exchange; MMF, mycophenolate mofetil; RTX, rituximab; TOF, tofacitinib.

**Table 4:** Timing of new diagnosis with PPF after treatment initiation in patients with PM/DM-ILD.

	>6 mo	6–12 mo	12–24 mo	24–36 mo	>60 mo
Anti-MDA5-Ab negative (n = 9), *n* (%)	2 (22.2)	1 (11.1)	1 (11.1)	3 (33.3)	2 (22.2)
Anti-MDA5-Ab positive (n = 5), *n* (%)	5 (100)				

PPF, progressive pulmonary fibrosis; PM/DM, polymyositis/dermatomyositis; ILD, interstitial lung disease; MDA5-Ab, melanoma differentiation-associated gene 5 antibody; mo, months.

**Table 5:** Comparison of chest HRCT findings regarding PPF between the anti-MDA5 antibody-positive and antibody-negative groups.

Chest HRCT Findings of PPF		Anti-MDA5-Ab Negative (*n* = 9)	Anti-MDA5-Ab Positive (*n* = 5)	*p*-Value
Increased extent or severity of traction bronchiectasis and bronchiolectasis	n (%)	8 (88.9)	5 (100)	1
	months	13.9 (7.2–19.5)	3.3 (0.3–5.2)	0.016
New ground-glass opacity with traction bronchiectasis	n (%)	7 (77.8)	5 (100)	0.51
	months	14.6 (6.0–32.3)	3.3 (0.3–5.2)	0.023
New fine reticulation	n (%)	6 (66.7)	3 (60)	1
	months	15.8 (4.9–42.1)	4.8 (3.3–5.7)	0.16
Increased extent or increased coarseness of reticular abnormality	n (%)	6 (66.7)	2 (40)	0.58
	months	14.2 (9.5–33.6)	5.2 (4.8–5.7)	0.067
New or increased honeycombing	n (%)	3 (33.3)	0 (0)	0.26
	months	40 (33.9–71.6)		
Increased lobar volume loss.	n (%)	1 (11.1)	2 (40)	0.51
	months	6	3.1 (0.4–5.7)	0.54

The laboratory markers are presented as median (interquartile range). The *p*-values were estimated using Fisher’s exact test or Mann–Whitney U test. HRCT, high-resolution computed tomography; PPF, progressive pulmonary fibrosis; MDA5, melanoma differentiation-associated gene 5; Ab, antibody.

## Data Availability

The data presented in this study are available on request from the corresponding author due to the need to maintain patient privacy.

## Supplementary Materials

The following supporting information can be downloaded at: https://www.mdpi.com/article/10.3390/jcm14051601/s1, Table S1: Two-year changes in %DLCO after initiation of treatment in PPF cases. Table S2: Comparison of chest HRCT findings regarding PPF between the anti-MDA5 antibody-positive and antibody-negative A/S-ILD groups.

## Abbreviations

The following abbreviations are used in this manuscript:

PM	polymyositis
DM	dermatomyositis
IIMs	idiopathic inflammatory myopathies
ILD	interstitial lung disease
ARS	Aminoacyl-tRNA synthetase
MDA5	melanoma differentiation-associated gene 5
TIF1-γ	transcriptional intermediary factor 1-γ;
GCs	glucocorticoids
IVCY	intravenous cyclophosphamide
PF-ILD	progressive fibrosing ILD
HRCT	high-resolution computed tomography
PPF	progressive pulmonary fibrosis
A/S	acute/subacute
DLCO	diffusion capacity of the lung for carbon monoxide
AaDO_2_	alveolar-arterial oxygen gradient
FVC	forced vital capacity
GGO	ground-glass opacity
CSA	cyclosporin-A
TAC	Tacrolimus
MPDN	methylprednisolone
PE	plasma exchange
