# Supplementary material for: Comparison of Chest High-Resolution Computed Tomography Findings in Patients with Anti-Melanoma Differentiation-Associated Gene 5 Antibody-Positive and Antibody-Negative Progressive Pulmonary Fibrosis with Polymyositis/Dermatomyositis
Source: J Clin Med. 2025 Feb 27;14(5):1601. doi: 10.3390/jcm14051601 (PMC11900237; doi:10.3390/jcm14051601)
Supplement: Supplementary file 1 [file jcm-14-01601-s001.zip › jcm-3448455-supplementary.pdf]

Supplementary Table S1. Two-year changes in %DLCO after initiation of treatment in PPF cases.

Case	Antibody	Pre-treatment	6 months	1 year	18 months	2 year
1	Anti-MDA5-Ab positive	36.7	27	45.2	37.1	
2	Anti-MDA5-Ab positive	49.6	37.8	66.1		
3	Anti-MDA5-Ab negative	52.4		62		54.7
4	Anti-MDA5-Ab negative	35.7	42	39.4		42.2
5	Anti-MDA5-Ab negative	27.5	46.7	69.3		52.9
6	Anti-MDA5-Ab negative	20.6		38.5		43
7	Anti-MDA5-Ab negative	23.2		23.9		37.3
8	Anti-MDA5-Ab negative	44.9		63.4		

Values represent the percentage of predicted DLCO (%DLCO) measured at different time points (Pre-treatment, 6 months, 1 year, 18 months, and 2 years) after treatment initiation. DLCO, diffusing capacity of the lungs for carbon monoxide; PPF, progressive pulmonary fibrosis; Anti-MDA5-Ab: anti-melanoma differentiation-associated gene 5 antibody.

Supplementary Table S2. Comparison of chest HRCT findings regarding PPF between the anti-MDA5 antibody-positive and antibody-negative A/S-ILD groups.

**Chest HRCT findings of PPF**		**Anti-MDA5-Ab negative A/S-ILD (n=6)**	**Anti-MDA5-Ab positive A/S-ILD (n=5)**	***P***
Increased extent or severity of traction bronchiectasis and bronchiolectasis	n (%)	5 (83.3)	5 (100)	1
	months	10.7 (3.7–22.7)	3.3 (0.3–5.2)	0.047
New ground-glass opacity with traction bronchiectasis	n (%)	6 (100)	5 (100)	-
	months	15.8 (4.9–42.1)	3.3 (0.3–5.2)	0.036
New fine reticulation	n (%)	6 (100)	3 (60)	0.18
	months	15.8 (4.9–42.1)	4.8 (3.3–5.7)	0.16
Increased extent or increased coarseness of reticular abnormality	n (%)	4 (66.7)	2 (40)	0.57
	months	11.9 (7.2–57.0)	5.2 (4.8–5.7)	0.105
New or increased honeycombing	n (%)	1 (16.7)	0 (0)	1
	months	71.6		
Increased lobar volume loss	n (%)	1 (16.7)	2 (40)	0.55
	months	6	3.1 (0.4–5.7)	0.54

The laboratory markers are presented as median (interquartile range). The *P*-values were estimated using Fisher's exact test or Mann–Whitney U-test.

HRCT, high-resolution computed tomography; PPF, progressive pulmonary fibrosis; MDA5, melanoma differentiation-associated gene 5; Ab, antibody; A/SILD, acute/subacute interstitial lung disease.
